# The Protective Effects of *Nigella sativa* and Its Constituents on Induced Neurotoxicity

**DOI:** 10.1155/2015/841823

**Published:** 2015-10-29

**Authors:** Mohammad Reza Khazdair

**Affiliations:** Pharmaceutical Research Center and Department of Physiology, School of Medicine, Mashhad University of Medical Sciences, Mashhad 9177948564, Iran

## Abstract

*Nigella sativa* (*N. sativa*) is an annual plant and widely used as medicinal plant throughout the world. The seeds of the plant have been used traditionally in various disorders and as a spice to ranges of Persian foods. *N. sativa* has therapeutic effects on tracheal responsiveness (TR) and lung inflammation on induced toxicity by Sulfur mustard. *N. sativa* has been widely used in treatment of various nervous system disorders such as Alzheimer disease, epilepsy, and neurotoxicity. Most of the therapeutic properties of this plant are due to the presence of some phenolic compounds especially thymoquinone (TQ), which is major bioactive component of the essential oil. The present review is an effort to provide a comprehensive study of the literature on scientific researches of pharmacological activities of the seeds of this plant on induced neurotoxicity.

## 1. Introduction

*Nigella sativa* L. (*N. sativa*) is belonging to Ranunculaceae family; it is an annual herbaceous plant widely grown in the Mediterranean countries, Western Asia, Middle East, and Eastern Europe. The* N. sativa* seeds have been added as a spice to range of Persian foods such as bread, pickle, sauces, and salads [1]. The active constituents of* N. sativa*, principally thymoquinone (TQ), have potential therapeutic properties; they exhibited the anti-inflammatory effects on several inflammatory disorders including encephalomyelitis, colitis, Edema, and arthritis through suppression of prostaglandins and leukotrienes as inflammatory mediators [1].

*N. sativa* seeds in the Middle East, India, and Northern Africa are used traditionally for the treatment of asthma, cough, bronchitis, headache, rheumatism, fever, and influenza. Antihistaminic, antidiabetic, and anti-inflammatory activities of* N. sativa* also were showed [2]. The protective effect of* N. sativa* seeds against lead acetate-induced liver toxicity in male rats was demonstrated [3].

The therapeutic effects of* N. sativa* on tracheal responsiveness (TR) and lung inflammation on guinea pigs induced toxicity by Sulfur mustard were examined [4].* N. sativa* oil extract significantly improved the clinical symptoms in patients with allergic diseases such as bronchial asthma, allergic rhinitis [5].

Central analgesic effects of methanol and aqueous extract of* N. sativa* were evaluated using hot-plate test and pressure test. Reaction time in the hot-plate test and pressure tests are significantly induced by both of extracts [6].

*N. sativa* is well-known for its potent antioxidative effects [2] and also demonstrated that* N. sativa* seeds could preserve significantly the spatial cognitive in rats challenged with chronic cerebral hypoperfusion [7].

In addition,* N. sativa* can prevent the damage of spatial memory after scopolamine administration and reduced the acetylcholinesterase (AChE) activity as well as oxidative stress of the brain tissue in rats [8].

Chemical composition of* N. sativa* seeds includes oil, protein, carbohydrate, fiber, and saponin. The fixed oil chemical compositions of* N. sativa* are linoleic acid, oleic acid, palmitic acid, arachidic acid, eicosadienoic acid, stearic acid, linoleic acid, and myristic acid [9].

The major phenolic compounds of* N. sativa* seeds are p-cymene (37.3%), thymoquinone (TQ) (13.7%), carvacrol (11.77%), carvone (0.9%), and thymol (0.33%) [1, 10, 11].

In the present review work, it was aimed to highlight the possible beneficial effects of* N. sativa* and its constituents on induced neurotoxicity.

## 2. Antineurodegenerative Effects

### 2.1. *N. sativa* and Thymoquinone

The effects of* N. sativa* on induced neuronal injury by chronic toluene exposure in the frontal cortex and brain stem in rats were evaluated. Chronic toluene exposure (inhalation of 3,000 ppm toluene, in 8 hours/day) for 12 weeks caused severe degenerative changes including the following: cytoplasm was shrunk, cisternae of endoplasmic reticulum were dilated, mitochondria were swelled, and nuclear membrane broke down in neurons of the frontal cortex and brain stem. Histopathological investigation in the treated group with* N. sativa* (400 mg/kg body weight) once a day orally for 12 weeks after toluene exposure showed no histopathological changes of neurodegeneration in the frontal cortex and brain stem. In the* N. sativa* treated rats after chronic toluene exposure did not show any pathological changes in the nerve cells [12]. Similarly the effects of* N. sativa* and TQ on neurodegeneration in hippocampus after chronic toluene exposure in rats were studied.* N. sativa* (400 mg/kg body weight) and TQ (50 mg/kg body weight) once a day orally had protective effects in hippocampus after chronic toluene exposure and no histopathological changes of neurodegeneration in treatment group have been reported [13]. In addition, in the other study the protective effects of TQ (50 mg/kg body weight) once a day orally for 12 weeks after toluene exposure in frontal cortex have been reported [14].

## 3. Anti-Alzheimer Effects

### 3.1. Thymoquinone

Alzheimer disease (AD) is a neurodegenerative disorder and characterized by progressive brain atrophy, accumulation of cortical senile plaques which pathologically formed by aggregation of the 4.2-kD amyloid beta peptide (Ab), in the central nervous system [15].

The protective effects of TQ against different concentrations of Ab1–42 induced cell death in cultured hippocampal neurons (*in vitro*) were studied. Ab-induced increased cell death with dose dependent manner in hippocampal cell culture. Following exposure to different concentrations of TQ (0.1, 1, 10, and 100 nM) in hippocampal cells had no significant effect on the survival rate of hippocampal neurons. However, simultaneous use of TQ with Ab1–42 showed a significant improvement in cell survival. Ab1–42 induced generation of reactive oxygen species and potential depolarization in mitochondrial membrane which inhibited by TQ. Furthermore TQ inhibited Ab1–42 aggregation and restored synaptic vesicle recycling inhibition and also partially reversed the loss of spontaneous firing activity [16].

Similarly the effect of TQ (5 mg/kg/day p.o.) against transient forebrainischemia induced neuronal damage in the rat hippocampus was evaluated. Ischemia induced oxidative injury in rats demonstrated by remarkable increase in malondialdehyde and decrease significantly in catalase and superoxide dismutase (SOD) activities and glutathione (GSH) contents in the hippocampal tissue compared to the control group. Pretreatment of TQ significantly decreased the number of hippocampal cells' death (24% in TQ-treated group compared to 77% in ischemia group). In addition, pretreatment with TQ increased GSH contents, catalase, and SOD activities and also decreased the elevated malondialdehyde (MDA) levels. Furthermore, TQ and thymohydroquinone (THQ) inhibited lipid peroxidation induced by iron-ascorbate in hippocampal homogenate [17].

## 4. Other Phenolic Compounds

One of the therapeutic strategies for AD treatment is the use of acetylcholinesterase (AChE) inhibitors, the principal enzyme involved in the hydrolysis of acetylcholine (ACh) [18]. The AChE inhibitory effects of phenolic compounds including thymol, carvacrol, TQ, and THQ were evaluated. TQ showed the most strongest AChE inhibitory effect over the range of concentrations (Table 1) [19].

AChE inhibitory and antioxidant effects of some compounds including thymol and carvacrol were evaluated [20].

The protective effect of* Nigella sativa* and its constituents on neurodegeneration and Alzheimer disease (AD) was shown in Table 2.

## 5. Antiepileptic Effect

### 5.1. *N. sativa*

The effect of* N. sativa* oil (NSO) on changes of amino acid neurotransmitters (epilepsy) induced by pilocarpine (380 mg/kg, i.p.) was investigated. In hippocampus glycine and taurine decrease and aspartate increased significantly and also aspartate, glutamate, GABA, glycine, and taurine levels increased significantly in the cortex after pilocarpine injection.* N. sativa* oil (4 mL/kg) could not improve significantly the pilocarpine-induced abnormalities [21].

The effects of the aqueous seed extract of* N. sativa* on pentylenetetrazole (PTZ, 40 mg/kg b.w.) induced seizure on rats model were studied.* N. sativa* extract reduced locomotor activity, increased sleeping time, and impaired motor coordination. Resistance to convulsion in the pretreated animals with N. sativa extract was more than the control animals. Severity score decreased while duration of onset of seizure increased in* N. sativa* treated group. In addition,* N. sativa* inhibited picrotoxin (a GABAA antagonist) and the prolongation of seizure latency [26]. Anticonvulsant and antioxidant activities of NSO on PTZ (35 mg/kg, i.p.) induced kindling seizures in mice were investigated. NSO (12 mL/kg, p.o.) remarkably decreased the seizure score compared to valproate + PTZ mice and also protected against the convulsive behaviors and mortality induced by PTZ. In addition NSO remarkably increased the GSH levels and decreased the MDA level compared to the PTZ group [22].

In a clinical trial effects of aqueous extract of* N. sativa* (40 mg/kg) in reducing the frequency of seizures in childhood (13 years old) epilepsy were evaluated. All the patients (20 children) received extract (40 mg/kg) or placebo three times a day for a period of four weeks. The mean frequency of seizures was decreased significantly during the treatment with* N. sativa* extract [23].

### 5.2. Thymoquinone

Anticonvulsant activity of TQ in induced seizure by PTZ intracerebroventricular (i.c.v.) injection was investigated. The injection of TQ (200 and 400 *μ*mol, i.c.v.) prolonged the onset and reduced the duration of tonic-clonic seizures. The protective effect of thymoquinone against lethality was 45% and 50% in the 200 and 400 *μ*mol, respectively [24]. Similarly the effects of TQ on induced seizure models using (PTZ) and maximal electroshock (MES) were investigated. TQ (40 and 80 mg/kg) reduced the locomotor activity and have anticonvulsant activity through an opioid receptor-mediated increase in GABAergic tone [24].

A pilot, crossover clinical trial studied the effect of TQ (1 mg/kg) on children with refractory epilepsy. The patients (22 children) were assigned in two groups and received either TQ or placebo for a period of four weeks. The frequency of seizures in TQ group compared to placebo was significantly reduced [25].

The effect of* Nigella sativa* and TQ on epilepsy was shown in Table 3.

## 6. Anti-Parkinson Effects

### 6.1. *N. sativa*

Anti-Parkinson's activity of ethanolic extract of* N. sativa* seeds (EENS) in chlorpromazine (CPZ) induced neurotoxicity in animal model was suggested.

Chlorpromazine (3 mg/kg i.p.) induced catalepsy which is a widely accepted animal model for Parkinson's disease. Ethanolic extract of* N. sativa* at doses of 200 and 400 mg/kg significantly reduced catalepsy compared with CPZ treated group. EENS (200 and 400 mg/kg) significantly reversed the amount of lipid peroxidation and reversed the increase in nitrite level compared to CPZ group. In addition administration of EENS (200 and 400 mg/kg) increased significantly the level of GSH compared with CPZ treated rats [27].

The effects of* N. sativa* hydroalcoholic seed extract orally used on muscle stiffness in perphenazine-induced muscle rigidity in adult male mice were evaluated.* N. sativa* (100 mg/kg) significantly improved the muscle rigidity score starting at the 40th minute, while animals treated with extract (50 mg/kg) had no significant difference with control group (received water). Moreover,* N. sativa* (200 mg/kg) significantly improved the muscle rigidity score starting at all times measured in comparison with control group [28].

### 6.2. Thymoquinone

The effect of TQ on behavioral, cellular abnormalities and markers of oxidative stress in unilateral intrastriatal 6-hydroxydopamine (6-OHDA) induced early Parkinson model of rat was evaluated. TQ pretreatment (5 and 10 mg/kg) remarkably enhanced turning behavior, prevented loss of substantia nigra pars compacta (SNC) neurons, and reduced level of MDA [29].

### 6.3. Carvacrol

Carvacrol (CAR) is a monoterpenic phenol found in* N. sativa*. The effects of CAR (10 mg/kg) on unilateral intrastriatal 6-hydroxydopamine induced Parkinson's disease, apomorphine-induced rotations, and also effects of CAR (10 mg/kg) on the level of stress oxidative markers in the midbrain were measured after 2 weeks.

CAR administration reduced the rotation number in lesioned rats. Also, CAR decreased the MDA and nitrite level and increased the antioxidant enzyme catalase, and it also has a protective effect against lipid peroxidation and free radicals synthesis [30].

The effect of* Nigella sativa* and constituents on Parkinson's disease was shown in Table 4.

## 7. Antioxidant Effects

### 7.1. *N. sativa*

Protective effect of* N. sativa* seed extracts on oxidative stress by STZ (60 mg/kg) induced diabetic rats was showed.* N. sativa* extract (200 mg/kg) increased the thiol content of hippocampus compared to untreated diabetic group. MDA content of hippocampus reduced significantly in* N. sativa* extracts (200, 400 mg/kg), in comparison to the untreated diabetic rats where the dose of 200 mg/kg was more effective to reduce oxidative stress in hippocampus of rats [31].

Pretreatment with NSO (0.048, 0.192 and 0.384 mg/kg) injected intraperitoneally immediately after reperfusion and administration was continued every 24 hours to 72 hours after induction of ischemia resulted in a significant decreased in MDA level compared with ischemic group [32].

Therapeutic effects of* N. sativa* hydroalcoholic extract in PTZ-induced repeated seizures on brain tissues oxidative damage were investigated. The time spent in target quadrant in Morris water maze (MWM) test and delay time to enter the dark in PTZ group was lower than control group, while* N. sativa* extract (400 mg/kg) increased them significantly.* N. sativa* extract (200 and 400 mg/kg) decreased the MDA concentration in hippocampal tissues and total thiol concentration in hippocampal in* N. sativa* extract (400 mg/kg) was increased compared to the PTZ group [33].

*N. sativa* has protective effects on hypothyroidism-induced oxidative damage in brain tissue by propylthiouracil (PTU).* N. sativa* extract (400 mg/kg) and vitamin C (100 mg/kg) increased the time spent in target quadrant while reducing the time latency compared to the PTU group. The serum thyroxine concentrations in animals treated by* N. sativa* extract (100, 200, and 400 mg/kg) as well as by Vit C were higher than that of the PTU group [34].

### 7.2. Thymoquinone

Pretreatment animals with TQ (2.5, 5, and 10 mg/kg, i.p.) immediately after reperfusion and continued administration after induction of ischemia were showed a significant decrease in MDA level compared with ischemic group [32].

Administration of lead acetate caused pathological disorder including degeneration of endothelial lining of brain blood vessels, chromatolysis and neuronal degeneration, ischemic brain infarction, degeneration of hippocampal and cerebellar neurons, microglial reaction, and neuronophagia in rat model but control and TQ (20 mg/kg b.w) treated rats showed normal brain histology. In addition, coadministration of TQ with lead acetate significantly decreased the incidence of lead acetate-induced pathological lesions [35].

### 7.3. Carvacrol

The protective effects of CAR on cerebral ischemia-reperfusion injury in a middle cerebral artery occlusion mouse model were investigated. CAR (50 mg/kg) remarkably reduced infarct volume and also improved neurological deficits after 75 minutes of ischemia and 24 hours of reperfusion. Furthermore, posttreatment with (CAR, i.c.v.) reduced infarct volume at 6 hours after reperfusion [36].

Methotrexate (MTX) is generally used for the treatment of malignancies, which has many systematic side effects. Protective effects of CAR (73 mg/kg, i.p.) and pomegranate (POM) (225 mg/kg) against MTX induced oxidative stress and inflammation were investigated. In the MTX + CAR group, total oxidant status (TOS), MDA, IL-1 *β*, and TNF-*α* levels were decreased significantly while total antioxidant status (TAS) was increased in comparison to the MTX group. In the MTX-POM group, MDA, IL-1 *β*, and TNF-*α* levels were decreased significantly and there was not a significant change in TAS and TOS levels in comparison to the MTX group. In addition, TNF-*α* level was lower in the MTX + CAR group compared to the MTX + POM group but other parameters (TAS, TOS, MDA, and IL-1 *β*) were similar in both groups [37].

The protective effect of* Nigella sativa* and its constituents on oxidative stress was shown in Table 5.

## 8. Clinical Applications

In a clinical trial forty-two patients with rheumatoid arthritis (RA) were assigned to intervention group receiving capsules of* Nigella sativa* oil (500 mg) and placebo each day for 8 weeks. Whole blood levels of oxidative stress parameters were measured at baseline and end of the trial. The serum level of IL-10 was increased and serum MDA and NO significant reduced in the* N. sativa* group compared with baseline.* N. sativa* improved inflammation and reduced oxidative stress in patients with RA [38].

Similarly forty healthy volunteers were divided randomly into two groups, treatment with capsules of* N. sativa* (500 mg) and placebo (500 mg) twice daily for nine weeks.* N. sativa* treatment group enhanced memory, attention, and cognition compared to the placebo group [39].

In a clinical study* N. sativa* oil extract, administered in a dose of 40 mg/kg, which significantly improved the clinical symptoms in patients with allergic diseases such as bronchial asthma, allergic rhinitis, and atopic eczema and all extract of* N. sativa* oil except high doses of 80 mg/kg in children did not have adverse effects [5].

In another clinical study 48 healthy adolescent aged between 14 and 17 years were randomly divided into two groups: A: 500 mg placebo and B: 500 mg* N. sativa* once daily for 4 weeks. All healthy adolescents were evaluated for mood with Bond-Lader scale, anxiety with State-Trait Anxiety Inventory (STAI), and cognition with modified California verbal learning test-II (CVLT-II), at the beginning and the end of study.* N. sativa* decreased anxiety, stabilized mood, and modulated cognition in the end of study [40].

## 9. Conclusion

This review article summarized a variety of studies in order to find out the effects of* N. sativa* and its constituents especially its main constituents (TQ) on induced neurotoxicity. The results of numerous studies have shown that the* N. sativa* seed exhibits beneficial effects in different central nervous system disorders including memory impairment, epilepsy, and neurotoxicity. Furthermore, based on the current review, it is concluded that* N. sativa* seed and main constituents through inhibition of AChE activity and increasing the GABAergic tone and particularly antioxidant effects improved nervous system diseases.

## Figures and Tables

**Table 1:** The AChE inhibitory effects of some phenolic compounds.

Phenolic compounds	EC_50_
Thymol	0.74
Carvacrol	0.063
TQ	0.14
THQ	0.04
Galantamine	0.00025

(EC_50_): the effective concentration causing 50% of maximum response in each experiment was measured using the log concentration-response curve. Galantamine (a natural AChE inhibitor) in Table 1 was taken from the study of Jukic et al. [19].

**Table 2:** The protective effect of *Nigella sativa *and its constituents on neurodegeneration and Alzheimer disease (AD).

Drug	Dose	Result	References
*N. sativa*	400 mg/kg	No histopathological changes observed in the frontal cortex and brain stem	[12]

*N. sativa* and TQ	400 mg/kg and 50 mg/kg	No histopathological changes observed in hippocampus	[13]

TQ	50 mg/kg	Protective effects in frontal cortex	[14]

TQ	(0.1, 1, 10, and 100 nm)	Significant improvement in cell survival	[16]

TQ	5 mg/kg/day	Increased GSH contents, catalase, and SOD activities and also decreased the elevated of MDA	[17]

**Table 3:** The effect of *Nigella sativa* and TQ on epilepsy.

Drug	Dose	Result	References
*N. sativa* oil	4 mL/kg	Did not improve significantly the pilocarpine-induced abnormalities	[21]

*N. sativa* oil	12 mL/kg	Decreased the seizure score, protected against the convulsive behaviors and mortality, increased the GSH levels, and decreased the MDA level compared to the PTZ group	[22]

*N. sativa*	40 mg/kg	Mean frequency of seizures was decreased	[23]

TQ	200 and 400 *μ*mol	Reduced the duration of tonic-clonic seizures	[24]

TQ	40 and 80 mg/kg	Reduced the locomotor activity	[24]

TQ	1 mg/kg	Reduced the frequency of seizures	[25]

**Table 4:** The protective effect of *Nigella sativa *and its constituents on Parkinson's disease.

Drug	Dose	Result	References
*N. sativa*	200 and 400 mg/kg	Reversed the extent of lipid peroxidation, reversed the increase in nitrite level, and increased the level of glutathione	[27]

*N. sativa*	100 and 200 mg/kg	Improved the muscle rigidity score	[28]

TQ	5 and 10 mg/kg	Prevented loss of SNC neurons and reduced level of MDA	[29]

Carvacrol	10 mg/kg	Decreased the MDA and nitrite level and increased the antioxidant enzyme catalase	[30]

**Table 5:** The protective effect of *Nigella sativa *and its constituents on oxidative stress.

Drug	Dose	Result	References
*N. sativa*	200 mg/kg	Increased the thiol content and reduced the level of MDA in hippocampus	[31]

NSO	0.048, 0.192, and 0.384 mg/kg	Significantly decreased the MDA level	[32]

*N. sativa*	200 and 400 mg/kg	Increased the time spent in target quadrant and delay time to enter the dark compared to the PTZ group	[33]

*N. sativa*	400 mg/kg	Increased the time spent in target quadrant, while reduced the time latency compared to the PTU group	[34]

TQ	2.5, 5, and 10 mg/kg	Decrease the MDA level compared with ischemic group	[32]

TQ	20 mg/kg	Decreased the incidence of lead acetate-induced pathological lesions	[35]

Carvacrol	50 mg/kg	Reduced infarct volume and also improved neurological deficits	[36]

Carvacrol	73 mg/kg	TOS, MDA, IL-1 *β*, and TNF-*α* levels were decreased significantly while TAS was increased in comparison to the MTX group	[37]
